# Data-Driven Subtyping of Executive Function–Related Behavioral Problems in Children

**DOI:** 10.1016/j.jaac.2018.01.014

**Published:** 2018-04

**Authors:** Joe Bathelt, Joni Holmes, Duncan E. Astle, Centre for Attention Learning and Memory (CALM) Team, Joni Holmes, Susan Gathercole, Duncan Astle, Tom Manly, Rogier Kievit

**Affiliations:** MRC Cognition and Brain Sciences Unit, University of Cambridge, UK

**Keywords:** executive function, childhood, nosology, structural imaging

## Abstract

**Objective:**

Executive functions (EF) are cognitive skills that are important for regulating behavior and for achieving goals. Executive function deficits are common in children who struggle in school and are associated with multiple neurodevelopmental disorders. However, there is also considerable heterogeneity across children, even within diagnostic categories. This study took a data-driven approach to identify distinct clusters of children with common profiles of EF-related difficulties, and then identified patterns of brain organization that distinguish these data-driven groups.

**Method:**

The sample consisted of 442 children identified by health and educational professionals as having difficulties in attention, learning, and/or memory. We applied community clustering, a data-driven clustering algorithm, to group children by similarities on a commonly used rating scale of EF-associated behavioral difficulties, the Conners 3 questionnaire. We then investigated whether the groups identified by the algorithm could be distinguished on white matter connectivity using a structural connectomics approach combined with partial least squares analysis.

**Results:**

The data-driven clustering yielded 3 distinct groups of children with symptoms of one of the following: (1) elevated inattention and hyperactivity/impulsivity, and poor EF; (2) learning problems; or (3) aggressive behavior and problems with peer relationships. These groups were associated with significant interindividual variation in white matter connectivity of the prefrontal and anterior cingulate cortices.

**Conclusion:**

In sum, data-driven classification of EF-related behavioral difficulties identified stable groups of children, provided a good account of interindividual differences, and aligned closely with underlying neurobiological substrates.

Executive functions (EFs) are a collection of cognitive processes that help to regulate thoughts and behavior. They are critically involved when we make plans, solve problems, and attain goals.^1^ Better EF is linked to many positive outcomes^2^ such as greater success in school,^3^, ^4^ better physical and mental health,^5^, ^6^ and better overall quality of life.^7^ In contrast, deficits in EF are associated with slow school progress,^8^ difficulties in peer relationships,^9^ and poor employment prospects.^10^ Behaviorally, EF deficits may manifest as distractibility, fidgetiness, poor concentration, chaotic organization of materials, and trouble completing work. EFs comprise multiple dissociable domains,^11^ and children may show different profiles of strength and weaknesses across these domains. This is also the case for diagnostic groups that are defined by behaviors that relate to EF, for example, attention-deficit/hyperactivity disorder (ADHD).^12^ In fact, difficulties in EF have been associated with several common neurodevelopmental disorders, including ADHD,^13^ autism spectrum disorder (ASD),^14^ and dyslexia.^15^ Despite the strong association between EF and outcomes highly relevant to children’s development, the heterogeneity of EF difficulties across children makes it difficult to devise effective intervention strategies and to investigate etiological mechanisms. The aim of the current study was to use a data-driven approach to identify groups of children with similar profiles of EF-associated behavioral problems and to relate these profiles to differences in white matter connectivity.

Data-driven subgrouping can provide the practical advantage of clearly defined groups of children with highly similar behavioral problems. In turn, this may help with identifying the pathophysiological mechanisms associated with those shared difficulties. The current study used a data-driven community clustering approach to group children by the similarity of their behavioral problems. In contrast to widely used factor-analytic approaches that aim to reduce measured variables to a smaller set of latent factors (e.g., grouping questionnaire items that relate to hyperactivity or inattentiveness), the clustering approach that we used groups children by similarities in their behavioral ratings. This alternative approach is made possible by recent advances in network science methods. Most clustering algorithms necessitate a priori assumptions, such as the geometrical properties of the cluster shape, the tuning of some parameters, or setting the number of desired clusters. These assumptions are difficult to make, but network science provides a possible solution. Network science is the study of complex networks, which represent relationships among data as a network of nodes connected by edges. This methodological approach provides a mathematical tool for quantifying the organization of networks and the relationships among the nodes within them.^16^ Defining subdivisions of highly connected nodes within a network, so-called communities, is an area of network science that has received considerable attention, as it applies to many real-world problems.^17^ In the case of psychometric data, the network can represent the similarity of scores between participants. Community detection makes it possible to define subgroups of participants that are most similar while being as distinct as possible from other subgroups. Our aim was to identify clusters in a large sample of children, according to the similarity of their EF-related behavioral problems, using a community detection approach based on the Conners questionnaire. This scale is routinely administered in health care and educational settings in many clinics in the United Kingdom.

We applied the data-driven clustering approach in a large sample of children (N = 442) identified as having problems in attention, learning, and/or memory by educational and clinical professionals working in various specialist children’s services. This sample includes common, complex, and comorbid cases of behavioral and cognitive difficulties. Many of these children would not be recruited by studies that use strict exclusory criteria to identify rarer selective cases, but are routinely seen by specialist clinicians and educators. This large heterogeneous group of children provides a perfect dataset for testing data-driven grouping methods. Moreover, understanding the profiles of EF-associated behavioral difficulties in children currently receiving the attention of these specialists may provide useful information for practitioners and may shift research focus toward these relevant behavioral profiles.

An additional aim was to relate the profiles of EF-associated behavioral problems to potential biological mechanisms. We explored differences in white matter connectivity between the groups identified through the community detection. White matter maturation is a crucial process of brain development that extends into the third decade of life,^18^ and which relates closely to cognitive development.^19^, ^20^, ^21^ It is thought to support cognitive development through better communication and integration among brain regions, particularly over longer distances,^22^ Accordingly, the brain can be modeled as a network of brain regions connected by white matter, commonly referred to as a connectome. Brain regions vary in the number of their connections— their node degree—which gives an indication of their importance for the network.^23^ To explore which brain regions were most closely linked to the behavioral profiles identified through community clustering, we used a multivariate dimension-reduction technique called partial least squares (PLS).^24^ In our analysis, PLS defined brain components that maximally distinguished the behaviorally defined groups.

## Methods

### Participants

**Figure 1:**
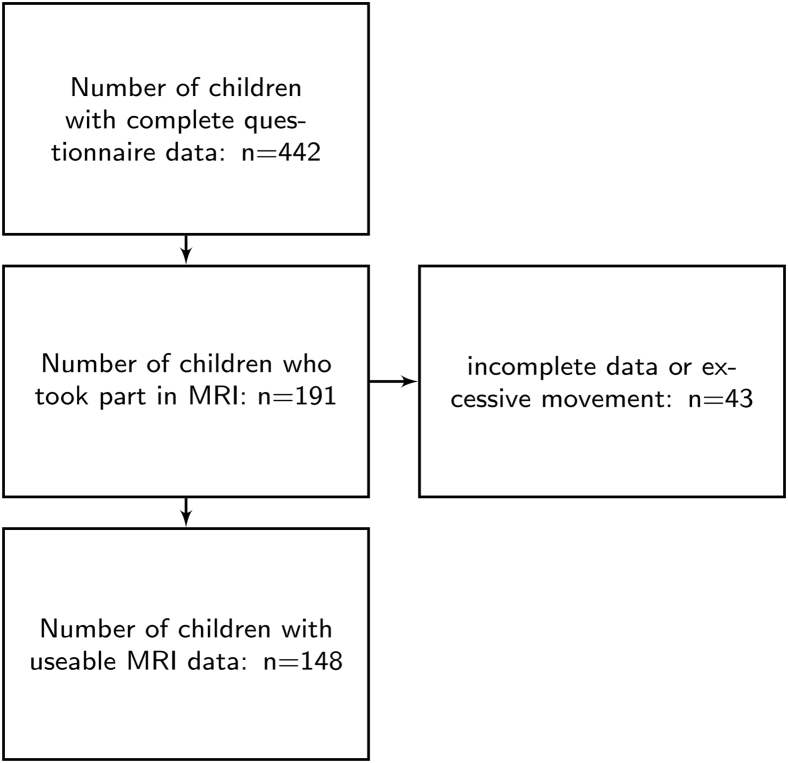
Overview of Data Included in Behavioral and Connectome Analysis ***Note:****MRI = magnetic resonance imaging*.

The sample consisted of ratings on 442 children (age: mean = 110.51 months; SE = 1.24; range = 62−215; 295 male). The proportion of boys was higher in this referred sample, in line with prevalence estimates of ADHD in the UK (ADHD: 4:1 male:female).^25^ Behavioral difficulties associated with ADHD were assessed using the Conners Parent Rating Short Form 3rd edition,^26^ herein referred to as “Conners 3.” The ratings were completed by parents or caregivers as part of a larger ongoing study at the Centre for Attention, Learning and Memory (CALM) at the MRC Cognition and Brain Sciences Unit, University of Cambridge. Children were recruited to the CALM research clinic on the basis of having problems in attention, learning, and/or memory that had come to the attention of a professional working in schools (e.g., special needs teacher) or specialist children’s community services (e.g., clinical or educational psychologists, speech and language therapists, or pediatricians). During the clinic visit, children completed a wide range of cognitive assessments, and their parents/caregivers filled in questionnaires about their child’s behavior. Children were also invited for magnetic resonance imaging (MRI) (see Figure 1 for attainment). The data reported here include those from 3 questionnaires and the MRI data. Exclusion criteria for referrals were significant or severe known neurological disorders, problems in vision or hearing that were uncorrected, or a native language other than English. This study was carried out in accordance with the Declaration of Helsinki and was approved by the local NHS Ethics committee (Reference: 13/EE/0157). Parents/caregivers provided written consent, and children gave verbal assent.

Some children in the broad sample of children referred for problems relating to attention, learning, and/or memory had received diagnoses through standard community services (see Table 1 for a breakdown of diagnoses). Among the children with a diagnosis, ADHD was the most common. Other diagnostic labels were rare. Therefore, diagnostic labels were grouped together. Primary diagnoses of dyslexia, dyscalculia, or dysgraphia were summarized as “learning deficits.” Primary diagnoses of autism spectrum disorder, autism, or Asperger syndrome were summed as “ASD.” Other labels, such as OCD, depression, anxiety, or developmental delay occurred only in a few individuals and were grouped as “other.”

**Table 1:** Breakdown of Children by Pre-existing Diagnoses and Referral Routes

Diagnosis	Total	%
None	302	76.7
ADHD	61	15.6
Learning deficit	32	8.2
ASD	24	6.2
Other	23	5.9

Referrer	Total	%
SENCo	262	66.9
Pediatrician	82	21.0
Clinical psychologist	29	7.4
Speech and language Therapist	29	7.4
Specialist teacher	13	3.3
ADHD nurse practitioner	13	3.3
Educational psychologist	6	1.5
Family worker locality team	5	1.3
Child psychiatrist	2	0.5
Private tutor	1	0.3

Note: ADHD = attention-deficit/hyperactivity disorder; ASD = autism spectrum disorder; SENCo = special educational needs coordinator.

### Behavioral Analysis

#### Questionnaire Data

The Conners 3 scale^26^ is a parent questionnaire designed to assess behavioral difficulties associated with ADHD and related disorders. It is well validated, with good psychometric integrity (internal consistency: Cronbach’s α = 0.91 [range 0.85−0.94]; factorial validity: root mean square error of approximation [RMSEA] = 0.07 based on confirmatory factor analysis in a replication sample; for details, see Conners^26^). Questionnaire items are summarized into 6 subscales (Inattention, Hyperactivity/Inattention, Learning Problems, Executive Function, Aggression, Peer Problems), and a total ADHD score is also derived. T scores of 60 and above are indicative of clinical levels of problems. A high proportion of children in the sample had scored in this range on each of the subscales (Table 2).

**Table 2:** Scores on Each Scale of the Conners 3 Questionnaire (Inattention, Hyperactivity/Impulsivity, Learning Problems, Executive Function, Aggression, Peer Relationships) for the Entire Sample

Scale	Mean	SD	Min	Max	*T* > 60	*T* > 60%
Inattention	79.74	11.955	40	90	398	90.0
Hyperactivity/Impulsivity	72.87	16.338	40	90	315	71.3
Learning Problems	75.95	11.912	42	90	390	88.2
Executive Function	73.81	12.906	40	90	363	82.1
Aggression	62.95	17.268	34	90	205	46.4
Peer Relationships	71.94	17.973	44	90	290	65.6

Note: The last 2 columns indicate the total number and the percentage of children in the sample with *T* scores in the clinical range on each scale. Max = maximum; Min = minimum.

The Conners 3 also contains 2 validity scales to detect response bias (that is, the rater tries to convey an overly positive or negative impression to secure a certain outcome).^26^ The validity scales indicated a possibly overly negative response style for 80 responses. Highly negative scores may indicate extreme problems in the rating domains or a negative bias of the rater, which may overestimate the child’s difficulties. Analyses were carried out including and excluding ratings with high negative impression scores.

The Behavioral Rating Inventory of Executive Function (BRIEF) is a questionnaire about behaviors associated with EF problems for parents of children and adolescents 5 to 18 years of age.^27^ There are 8 subscales measuring behavior problems related to inhibition, shifting, emotional control, initiation, working memory, planning/organizing, organization of materials, and monitoring.

The Strengths and Difficulties Questionnaire (SDQ) is a parent-rated scale for children and adolescents 8 to 16 years of age. It provides ratings for emotional symptoms and prosocial behavior as well as scores for problems related to behavioral conduct, hyperactivity/inattention, and peer relationships.

### Community Detection

**Figure S1:**
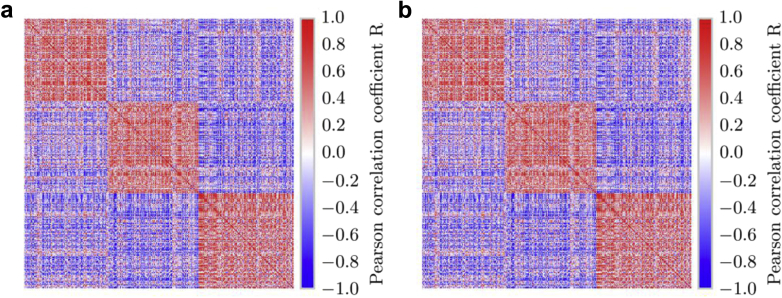
(A) Community-Grouped Adjacency Matrix Based on the Community Clustering Algorithm Used in the Main Analysis. (B) Community-Grouped Adjacency Matrix Based on the Kernighan−Lin Algorithm

**Figure S2:**
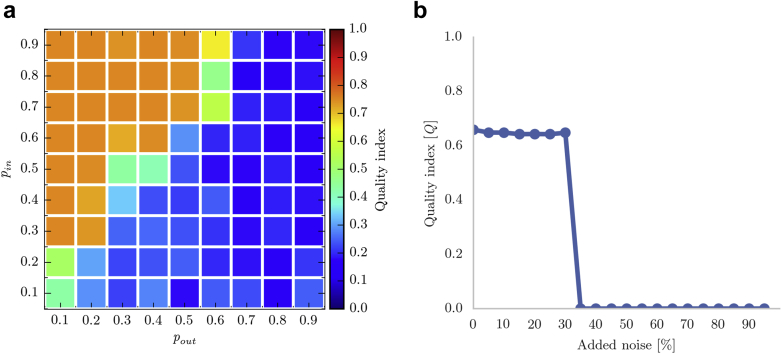
Results of Robustness Testing ***Note:****(a) Quality indices of consensus clustering using simulated networks with varying levels of within (*$p_{in}$*) and between (*$p_{out}$*) connections probabilities. High within-cluster and low between-cluster connectivity led to high separation of clusters with consensus clustering, that is, high quality indices. (b) Consensus clustering using the empirical child-by-child network of Conners 3 correlations with varying levels of added noise. The 3-cluster solution could be reconstructed up to 30% of added Gaussian noise. At a higher level of noise, no clustering solution could be obtained.*

**Figure S3:**
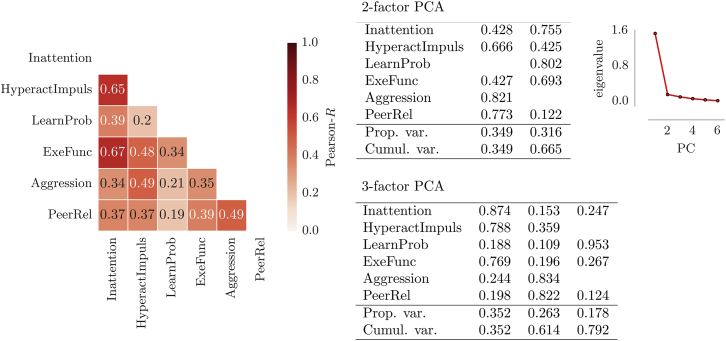
Principal Component Analysis (PCA) of the Conners 3 Scales ***Note:****The left panel shows the correlation matrix. The tables show the factor loadings and explained variance (prop. = proportional; cumul. = cumulative). The right figure shows the eigenvalues of each component (scree plot).*

Community detection is an optimization clustering method. Networks in the current analysis represented the child-by-child correlations across the 6 scales of the Conners 3 questionnaire. Questionnaire scales were used because the 4-point range of individual items was too limited to distinguish individuals. The community algorithm starts with each network node, namely, child, in a separate community and then iteratively parcellates the network into communities to increase the quality index (Q), which represents the segregation between communities with higher values indicating stronger segregation, until a maximum is reached. The current study used the algorithm described by Rubinov and Sporns^28^ as implemented in the Brain Connectivity Toolbox (https://sites.google.com/site/bctnet/) version of August 2016, which is an extension of the method described by Blondel *et al.*^29^ to networks with positive and negative edges. This algorithm is not deterministic and may yield different solutions at each run. To reach a stable community assignment, we applied the consensus clustering method described by Lancichinetti and Fortunato^30^ (see Figure S1, available online, for a comparison with an alternative algorithm). In short, an average community assignment over more than 100 iterations was generated. The community assignment was then repeated further until the community assignment did not change between successive iterations. The robustness of the community detection was tested with simulated networks with known community structure varying the probability of within-module versus between-module connections and adding random noise. The results indicated that the implementation of the algorithm could detect the community structure reliably over a range of these parameters (see Supplement 1 and Figure S2, available online). The analysis was implemented in Python 2.7.11. The code for the entire analysis is available online (https://github.com/joebathelt/Conners_analysis). A comparison exploratory factor analysis with principal component analysis is presented in the Supplement (see Figure S3, available online).

### Statistical Analysis

Groups defined by the community detection algorithm were compared on scales of the Conners 3 questionnaire. Shapiro−Wilk tests indicated that scores within groups deviated from normality assumptions.^31^ Group contrasts were therefore based on nonparametric Mann−Whitney *U* tests. The Bonferroni method was used to account for multiple comparisons. Statistical tests were carried out using Scientific Python (SciPy) version 0.17.0 implementation.^32^

#### Structural Connectome

The aim of this analysis was to explore whether the data-driven grouping was related to differences in brain structure. To this end, white matter connectivity of brain regions was estimated from diffusion-weighted images. Next, we used a multivariate, dimension-reduction technique to relate the white matter connectivity of brain regions to the group assignment.

#### Participant Sample for the Connectome Analyses

A subset of 191 families agreed to participate in the neuroimaging part of the study. A total of 43 scans were excluded for their poor quality, that is, incomplete scan data, visually identified movement artifact, maximum displacement in the diffusion sequence of more than 3mm as determined by FMRIB Software Library (FSL) eddy (see Figure 1 for an overview of attrition). The final sample consisted of 148 complete data sets (behavior, T1, diffusion-weighted images). The MRI sample did not significantly differ in age from the behavioral sample (MRI sample [months]: mean = 117.05, SD = 27.436, *t*(359) = 1.34, *p* = 0.181). The ratio of groups defined in the analysis of the behavioral sample was similar in the MRI subsample (MRI sample: C1: 0.36; C2: 0.33; C3: 0.30).

#### MRI Data Acquisition

Magnetic resonance imaging data were acquired at the MRC Cognition and Brain Sciences Unit, University of Cambridge. All scans were obtained on the Siemens 3 T Tim Trio system (Siemens Healthcare, Erlangen, Germany), using a 32-channel quadrature head coil. The imaging protocol relevant here consisted of 2 sequences: T1-weighted MRI and a diffusion-weighted sequence.

T1-weighted volume scans were acquired using a whole-brain coverage 3D Magnetization Prepared Rapid Acquisition Gradient Echo (MP RAGE) sequence acquired using 1-mm isometric image resolution. Echo time was 2.98 milliseconds, and repetition time was 2,250 milliseconds.

Diffusion scans were acquired using echo-planar diffusion-weighted images with an isotropic set of 60 noncollinear directions, using a weighting factor of b = 1,000 s **×** mm^−2^, interleaved with a T2-weighted (b = 0) volume. Whole-brain coverage was obtained with 60 contiguous axial slices and isometric image resolution of 2 mm. Echo time was 90 milliseconds, and repetition time was 8,400 milliseconds.

#### Structural Connectome Construction

**Figure 2:**
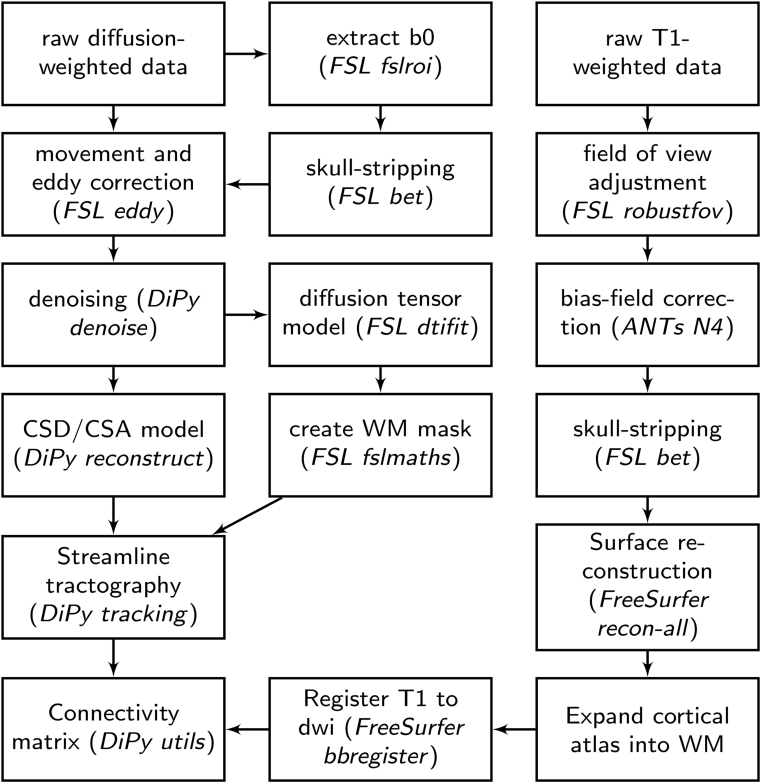
Overview of Processing Steps for Structural Connectome Estimation ***Note:****ANTs = Advanced Normalization Tools; DiPy = Diffusion Imaging in Python; FSL = FMRIB Software Library; WM = working memory. Other abbreviations are names of functions in the software packages mentioned.*

The white matter connectome reconstruction followed the general procedure of estimating the most probable white matter connections for each individual and then obtaining measures of fractional anisotropy (FA) between regions (Figure 2). White matter connectome reconstruction was carried out as previously described.^33^ Figure 2 provides an overview. The methodological details of the connectome construction are presented in Supplement 1, available online.

#### Statistical Analysis of Connectome Data

For the analysis of the connectome data, the node degree of each node in the network was calculated for each participant. Partial least squares (PLS) regression was used to identify the linear combination of brain areas that best explained group membership for the groups identified through community clustering. The PLS model was evaluated by fitting the model to a random selection of 60% of the data and evaluating the model fit in a test set of 40%. The root mean square error (RMSE) of a model based on the training data was significantly lower when assessed with the test data compared to randomly shuffled samples (10-fold cross-validated RMSE: mean = 0.35, SE = 0.025; permuted sample: mean = 0.81, SE = 0.018; permutation test: *p* = 0.002).

The contribution of brain regions to the PLS latent variables was evaluated in a bootstrap procedure in which 60% of the sample was randomly selected and the PLS model was fitted (1,000 permutations). The loading of brain regions onto PLS latent variables was expressed as the mean loading divided by the standard error across permutations.^24^ A Procrustes rotation was applied to align the factor across iterations of the permutation procedure. All procedures were implemented using sci-kit-learn functions v0.18.1 under Python v2.7.12.^34^

## Results

### Community Detection Indicates 3 Subgroups

The current study used graph theory to derive clusters of children with similar profiles across ratings on the Conners 3 questionnaire. The community detection algorithm in conjunction with consensus clustering arrived at a stable solution with 3 clusters. The quality index (Q = 0.55) indicated a good separation of the clusters (see Blondel *et al.*^29^ for quality indices of reference networks). A highly similar, 3-cluster structure was also detected when excluding participants with a high negative impression rating (Q = 0.59) and when randomly selecting one-half (Q = 0.6) or one-fourth (Q = 0.61) of the sample.

**Figure 3:**
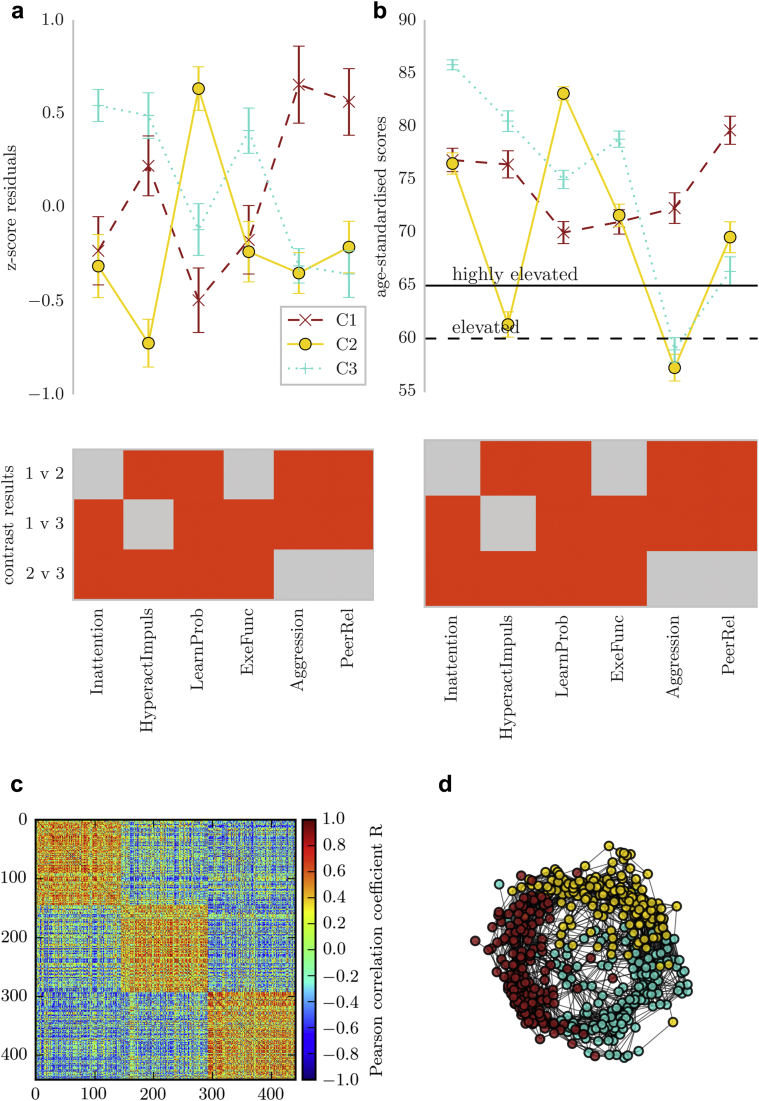
Overview of Community Clusters and Their Behavioral Profiles ***Note:****(a) Profile of ratings on the Conners 3 questionnaire in the 3 clusters indicated by the community detection algorithm. The top of the figure shows the mean of scores in each group with 2 standard errors. The scores represent residuals after regressing the effect of age. The bottom figure shows the results of groupwise contrasts on each scale. Red indicates a significant difference between groups after Bonferroni correction. (b) Comparison of the groups on scores standardized with reference to the normative data of the Conners 3 questionnaire. (c) Child-by-child correlation matrix of Conners 3 scores after ordering the matrix according to the cluster assignment indicated by community clustering. The order matrix shows a clear separation between the clusters. (d) Correlation matrix in a spring layout color-coded according to the cluster assignment indicated by community clustering. The spring layout representation shows clear spatial separation between the clusters. C1 = cluster 1 (inattention, hyperactivity/impulsivity/executive function); C2 = cluster 2 (learning problems); C3 = cluster 3 (aggression, peer relations).*

The cluster assignment resulted in roughly equal splits among the 3 clusters (cluster 1: 150 [33.93%]; cluster 2: 145 [32.80%]; cluster 3: 147 [33.25%]). There were significant differences on all subscales of the Conners 3 questionnaire between groups (Figure 3 and Table 3). Children in the clusters were characterized either by problems associated with cognitive control (C1: Inattention, Hyperactivity/Impulsivity/Executive Function), learning difficulties (C2: Learning Problems), or behavioral conduct problems (C3: Aggression, Peer Relations). There was no difference in age or gender distribution among the clusters (Table 4). Standardized scores indicated that the majority of children in the current sample scored in the elevated to highly elevated range across all Conners 3 subscales relative to age norms. The profiles based on scaled raw scores were also apparent when using the age-standardized scores (Figure 3b).

**Table 3:** Scales of the Conners 3 Questionnaire: Inattention, Hyperactivity/Impulsivity (HyperactImpuls), Learning Problems (LearnProb), Executive Function (ExeFunc), Aggression, Peer Relationship Problems (PeerRel)

Scale	Inattention/Hyperactivity/Executive Function (C1)		Learning Problems (C2)		Aggression/Peer Problems (C3)		1 vs. 2		1 vs. 3		2 vs. 3	
	Median	MAD	Median	MAD	Median	MAD	*U*	*p*	*U*	***p***	*U*	*p*
Inattention	0.11	0.810	0.71	0.404	0.01	0.959	4867	<.001	6487	<0.001	120.26	1.00
HyperactImpuls	–0.76	0.682	0.63	0.636	0.38	0.886	3149	<.001	9294	0.133	7321	<.001
LearnProb	0.90	0.524	–0.15	0.642	–0.58	0.876	3269	<.001	9051	0.052	4137	<.001
ExeFunc	–0.10	0.760	0.60	0.599	–0.13	0.953	5427	<.001	7027	<0.001	11782	1.00
Aggression	–0.59	0.497	–0.53	0.452	0.26	1.048	7649	.843	6885	<0.001	6871	<.001
PeerRel	–0.45	0.665	–0.56	0.571	0.67	0.938	8085	1.00	5.744	<0.001	7099	<.001

Note: All *p* values are Bonferroni corrected. MAD = median absolute deviance; U = Mann Whitney U statistic.

**Table 4:** Characteristics of Each Cluster

Group	N	Sex			Age, y			
		Male/Female	*χ*^*2*^	*p*^*a*^	Mean (SD)		*T*	*p*
C1	150	110/40	0.01	.934	9.28 (2.427)	1 vs. 2	–0.02	.981
C2	145	80/65	0.08	.771	9.28 (2.143)	1 vs. 3	1.03	.304
C3	147	115/32	0.04	.842	9.01 (2.023)	2 vs. 3	1.12	.263

Note: ^a^χ^2^ test in each group relative to the sex distribution in the whole sample. C1 = cluster 1 (inattention, hyperactivity/impulsivity/executive function); C2 = cluster 2 (learning problems); C3 = cluster 3 (aggression, peer relations).

^a^χ^2^ test in each group relative to the sex distribution in the whole sample.

Next, the prevalence of pre-existing diagnoses in each cluster was evaluated. Children with a diagnosis of ADHD were overrepresented in C1: Inattention, Hyperactivity/Impulsivity/Executive Function (see Table 5 for a breakdown of diagnoses per cluster, χ^2^(3,354) = 72.87, *p* < .001). Other diagnoses were equally distributed between the clusters; ASD: χ^2^(3,354) = 0.06, *p* = .971, Anxiety/Depression: χ^2^(3,354) = 0.54, *p* = .764, Learning Deficit: χ^2^(3,354) = 3.88, *p* = .144).

**Table 5:** Breakdown of Diagnoses in Each Cluster Identified Through Data-Driven Clustering

Diagnosis	C1	C2	C3	Total
ADHD	33	4	24	61
ASD	13	5	6	24
Learning deficit	3	22	7	32
Other	7	8	8	23
None	94	106	102	302
Total	150	145	147	442

Note: ADHD = attention-deficit/hyperactivity disorder; ASD = autism spectrum disorder; C1 = cluster 1 (inattention, hyperactivity/impulsivity/executive function); C2 = cluster 2 (learning problems); C3 = cluster 3 (aggression, peer relations).

### Subgroups Show Differences in Other Questionnaire Measures of Executive Function and Everyday Difficulties

**Figure 4:**
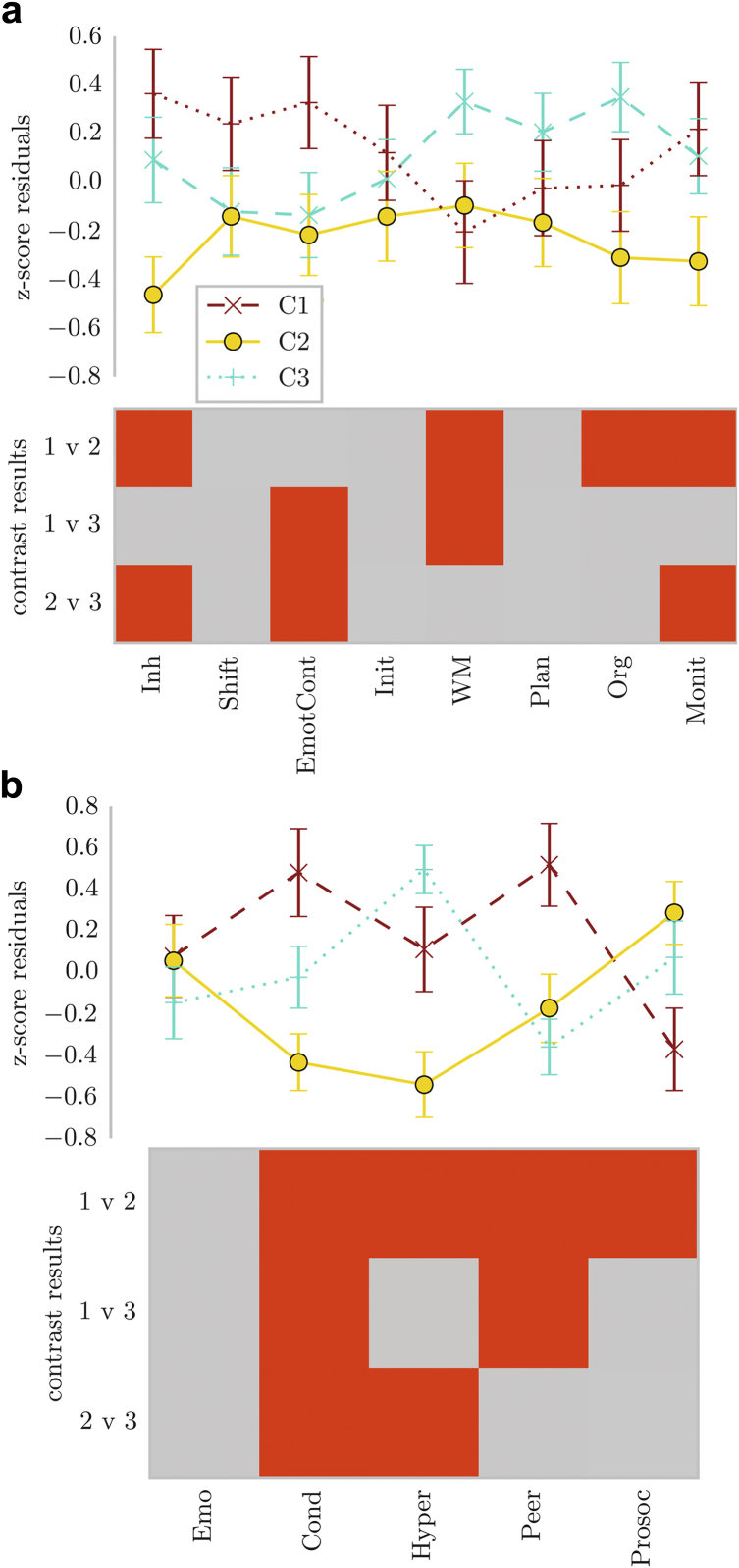
Profile of Ratings for Children in the Clusters Defined by Community Module Assignment on (a) a Questionnaire on Executive Function Difficulties (BRIEF) and (b) a Questionnaire on Strengths and Difficulties (SDQ) ***Note:****The lines indicate the mean of each group across the questionnaire scales, with error bars showing 2 standard errors around the mean. The bottom of each figure shows the binary outcome of t tests comparing the groups. Red indicates a significant result *(p*_corrected_<.05). after Bonferroni correction. Note that higher scores indicate a higher level of difficulties on each scale, apart from the Prosocial Behavior (Prosoc) scale, where high scores indicate more prosocial behavior. C1 = cluster 1 (inattention, hyperactivity/impulsivity/executive function); C2 = cluster 2 (learning problems); C3 = cluster 3 (aggression, peer relations); Cond = Conduct Problems; Emo = Emotional Problems; EmotCont = Emotional Control; Hyper = Hyperactivity; Inh = Inhibition; Init = Initiate; Monit = Monitoring; Org = Organization of Materials; Peer = Peer Problems; Prosoc = Prosocial Behavior WM = Working Memory.*

Next, the groups defined through community assignment based on Conners 3 data were compared on other questionnaire measures of behavioral problems linked to EF difficulties (BRIEF) and everyday behavioral problems (SDQ). A comparison of these measures indicated significant differences between the groups. For the BRIEF, children in Cluster 1 (Inattention/hyperactivity/Executive Function) had more problems with working memory. Children in Cluster 2 (Learning Problems) were rated as having fewer difficulties with inhibition and monitoring. Children in Cluster 3 (Aggression/Peer Problems) were also rated as having significantly higher problems in emotional control compared to the other groups (Figure 4a).

For the SDQ, children in Cluster 1 (Inattention/hyperactivity/Executive Function) were characterized by high ratings for hyperactivity compared to Cluster 2 (Learning Problems), but lower conduct and peer relationship problem ratings compared to Cluster 3 (Aggression/Peer Problems). Children in Cluster 2 (learning problems) received significantly lower ratings for problems related to hyperactivity. Children in Cluster 3 (Aggression/Peer Problems) received significantly higher ratings for conduct and peer relationship problems (Figure 4b).

### Data-Driven Grouping Leads to More Homogeneous Behavioral Profiles

The novel recruitment method of our sample, which includes children with specific, multiple, and no diagnoses, enabled us to explore the homogeneity of the behavioral profiles within established diagnostic categories by comparison with our data-driven groupings. For the statistical comparison, a random sample of 15 (65% of the smallest sample size) was drawn from all participants within a group, and correlations between their scales were calculated. This procedure was repeated 1,000 times to create a bootstrap sample of correlations. The correlations were averaged over the 3 data-driven groups and over the 4 diagnostic categories (ADHD, ASD, Learning Deficit, Other). The statistical comparison indicated that the difference between correlations in the data-driven groups and the diagnostic groups was significantly above 0, indicating higher correlations in the data-driven grouping (n = 1,000, mean = 0.23, SE = 0.001, *p* = 0.001). Crucially, the similarity was also significantly higher when comparing the data-driven groups to diagnostic groups on other questionnaires that were not used to inform the clustering algorithm (BRIEF: mean = 0.15, SE = 0.001, *p* = 0.001; SDQ: mean = 0.07, SE = 0.001, *p* = 0.026). This indicates that the data-driven grouping identified groups of children with more common profiles of behavioral symptomatology than we would expect to find in children grouped on the basis of more traditional diagnostic criteria.

### Subgroups Show Differences in the Structural Connectome

Next, we investigated the relationship between white matter connectivity and the groups defined through community clustering using partial least squares (PLS) regression. The first 3 PLS components explained 48% of variance in group membership (component 1: 21.23% [SD: 4.302]; component 2: 16.28% [SD: 5.944]; component 3: 10.57% [SD: 4.277], bootstrapped mean and standard deviation [SD] more than 1,000 permutations). Further components explained less than 5% of the variance and were therefore dropped from the analysis. Comparison of component loadings per group indicated significant lower loading of C1 (Inattention/Hyperactivity/Executive Function) compared to the other groups for PLS component 1, significantly higher loading in C1 (Inattention/Hyperactivity/Executive Function) compared to C3 (Aggression/Peer Problems) for PLS component 2, and significantly lower loading in C1 (Inattention/Hyperactivity/Executive Function) compared to C2 (Learning Problems) for PLS component 3.

**Figure 5:**
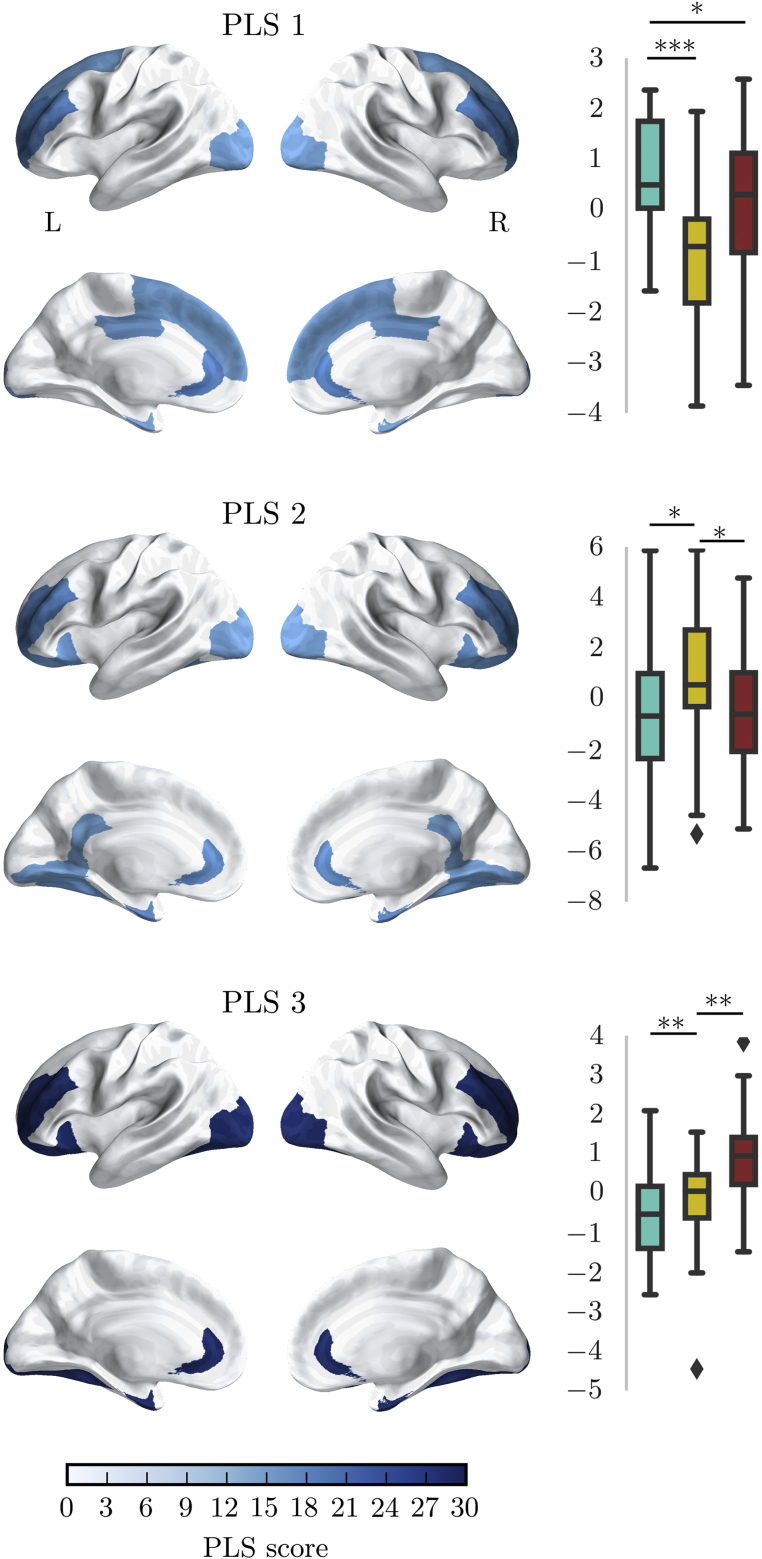
Relationship Between the Node Degree of Brain Regions in the Structural Connectome and Clusters Based on Conners 3 Responses ***Note:***The brain maps show the score of partial least squares (PLS) components for brain regions that most strongly distinguished the group (top 25%). PLS scores above 2 are considered to be significantly predictive. The graphs show the statistical comparison of groups on loadings for each component. *p < 0.05; **p < 0.01; ***p *< 0.001. C1 = cluster 1 (inattention, hyperactivity/impulsivity/executive function); C2 = cluster 2 (learning problems); C3 = cluster 3 (aggression, peer relations).*

There were differences in the brain areas that distinguished the groups. PLS 1, which distinguished between C1 (Inattention/Hyperactivity/Executive Function) and the other groups, loaded most heavily on the rostral middle frontal, superior frontal, lateral orbitofrontal, anterior cingulate, lateral occipital, and fusiform cortex (Figure 5). The second PLS component, which distinguished between C2 (Learning Problems) and C1 (Inattention/Hyperactivity/Executive Function), loaded the most on the rostral middle frontal, lateral orbitofrontal, anterior and posterior cingulate, and lateral occipital cortex. The third PLS component, which distinguished C3 (Aggression/Peer Problems) from the other groups, loaded on the lateral orbitofrontal, anterior cingulate, entorhinal and lateral occipital cortex, and also on connections of the right pallidum and putamen (Table 6).

**Table 6:** Loading of Partial Least Squares (PLS) Components on Subcortical Structures

Hemisphere	Structure	PLS 1	PLS 2	PLS 3
Left	Accumbens	0	0	0
	Amygdala	0	0	0
	Caudate	0	0	75
	Hippocampus	0	0	92
	Pallidum	0	0	0
	Putamen	0	0	80
	Thalamus	0	0	0
Right	Accumbens	0	0	0
	Amygdala	0	0	0
	Caudate	0	0	0
	Hippocampus	0	0	0
	Pallidum	0	0	0
	Putamen	0	0	0
	Thalamus	0	0	0

## Discussion

In this study, we used a data-driven clustering algorithm to group children according to their similarity on ratings of executive function (EF)−related behavioral problems. Among a large sample of children with common, complex, and comorbid behavioral problems, there exist distinct behavioral profiles. Three groups were identified: one with problems related to EF, inattention and hyperactivity; a second group with severe learning difficulties; and a third group with behavioral conduct problems. These groups were consistent in randomly selected subsets of the sample, and were reliably reproduced in simulated data with a known structure, even when adding considerable noise. The 3 behavioral profiles identified were evident in 2 additional parent rating scales that were not used to inform the original algorithm. Furthermore, comparison of white matter connectivity indicated that the data-driven groups were distinguished by connectivity of the lateral prefrontal and cingulate cortex

One of the subgroups was characterized by elevated symptoms of inattention, hyperactivity/impulsivity, and EF. This group was also rated as having increased difficulties with behaviors relating to working memory, organization, planning, and hyperactivity on 2 other rating scales that were not used as part of the clustering algorithm. This behavioral profile captures core problems associated with the ADHD diagnostic label, which is also marked by high levels of inattention and hyperactivity and EF problems.^13^, ^35^, ^36^, ^37^ A disproportionate number of children with an ADHD diagnosis were assigned to this cluster. However, this subtype was not synonymous with ADHD, as one-half of the children with an ADHD diagnosis were split across the other 2 clusters that were defined by markedly different behavioral profiles.

A second subgroup had more severe learning deficits relative to the other 2 groups. On other questionnaires, they were rated as having fewer problems with inhibition, attention, and other aspects of EF compared to children in the other clusters. However, their scores on these scales were in the elevated and clinical range when compared to age norms, indicating that they fall below age expected levels for attention and EF, but had less pronounced difficulties in these areas than children in the other clusters. Overall, this group displayed elevated symptoms of inattention and EF difficulties combined with fewer problems with hyperactivity/impulsivity. This profile resembles that described for the inattentive subtype of ADHD,^38^ but it should be noted that the most distinguishing feature of this group was pronounced learning difficulties rather than inattention.

A third subgroup was characterized by difficulties with aggression and peer relationships. Children in this group were also rated as having increased problems with behaviors related to emotional control and conduct on the 2 rating scales that were not used as part of the clustering algorithm. The distinction between groups with problems associated with either EF or behavioral conduct is reminiscent of the debate surrounding the overlap between ADHD and oppositional defiant disorder (ODD)/conduct disorder (CD). Some authors have argued for a high degree of overlap between these diagnostic groups,^14^ but evidence from genetic and imaging studies suggested distinct pathophysiological mechanisms.^39^, ^40^, ^41^ Consistent with these results, the current study shows that behavioral ratings of inattention/hyperactivity and aggression/peer relationship problems form distinct clusters.

These results demonstrate that data-driven clustering using a community detection algorithm can be used to characterize common and complex behavioral problems in children. The advantage of this approach is that groups identified through the algorithm display maximally homogeneous behavioral profiles. Greater behavioral homogeneity is likely to align more closely with potential biological mechanisms. Indeed, attempts to characterize subgroups based on brain function using similar community clustering techniques seem to converge on a similar distinction between children showing deficits with either cognitive control (C1, C2) or behavioral/emotional regulation (C3).^42^ In the current study, the exploratory analysis showed that our data-driven subgrouping was associated with underlying differences in structural connectivity between groups. The areas that distinguished the groups have been suggested to play a role in relevant behaviors, making it possible to formulate hypotheses about neurobiological mechanisms associated with the different behavioral profiles. For instance, the group characterized by problems relating to attention and EF showed differences in connectivity of the prefrontal, anterior cingulate cortex, and lateral occipital cortex. These differences in white matter connections of circuits related to inhibitory control,^43^ goal-directed behavior,^44^ and visual attention^45^ may play a role in the etiology of these behavioral problems. In contrast, children with a profile of problems relating to emotional regulation and peer relationships were distinguished from the other groups by differences in white matter connectivity of the rostrolateral prefrontal cortex, anterior cingulate cortex, pallidum, and putamen. These findings may imply a difference in integration between the prefrontal cortex and the basal ganglia system.^46^, ^47^ Brain differences associated with learning problems are more difficult to interpret because the majority of published studies focuses on much rarer specific learning problems, for example, dyslexia, dyscalculia, rather than general mechanisms of poor learning. Prefrontal and cingulate areas implicated in the current analysis may suggest the involvement of circuits involved in switching attention.^48^ Furthermore, ventral temporal areas have been implicated in both reading^49^ and mathematics,^50^ and may be related to mental imagery.^51^

It is important to be mindful of some caveats to our approach, and to the utility of data-driven grouping more generally. First, the grouping was based on parent-ratings, which have known limitations.^52^ Second, the grouping was based on just one behavioral checklist. We believe that it is important that these machine-learning approaches produce generalizable groups: that is, the groups must differ on other data not introduced to the algorithm. If groups can *only* be distinguished on the measures introduced to the machine learning, this suggests that there is not a genuine distinction between the groups, and instead the algorithm is overfitting. Our groupings differ on other questionnaires that were held out of the clustering process, but that are designed to tap similar constructs. Third, the diagnostic information was based on community practitioner assessments rather than diagnosis by a single evaluator within the research team. This approach is common with cohort studies that make use of community-reported diagnoses (e.g., Russell *et al.*^25^). Consequently, the diagnoses are reflective of children that typically present at secondary and tertiary services, and may be more informative about the children that these professionals routinely see. However, it is important to note that we cannot guarantee that these diagnoses reflect a diagnostic gold standard, which is sometimes sought for research purposes but which may not be reflected in community clinical diagnoses. Fourth, there were only a few cases with some diagnoses, for example, anxiety or ASD. Therefore, the study was not adequately powered to investigate homogeneity within these diagnostic groups.

In summary, clustering of similarities across behavioral problem identified 3 groups with distinct profiles of difficulties that related to inattention, learning, and peer relationships, respectively. These groups were also distinguished by the connectivity of circuits previously implicated in executive function and behavioral regulation, including the prefrontal cortex, cingulate cortex, and their subcortical connections. These findings act as an important proof of principle: data-driven profiling provides a means of distinguishing common and complex behavioral problems in children that relate closely to neurobiological mechanisms.

## Supplement 1

### Method

#### Formal Definition of the Quality Index Metric

We used a quality index that aims to maximise connections strength within modules while minimizing connection strength between modules. The metric described by Rubinov and Sporns^1^ as implemented in the Brain Connectivity Toolbox (https://sites.google.com/site/bctnet/) was used that incorporates positive and negative weights. The formal definition of the quality index from the original paper is as follows:

The connection between nodes i and j with positive weight is $w_{ij}^{+}\in (0,1]$ and with negative weight is $w_{ij}^{-}\in [-1,0)$. The total sum of all positive connections is $v^{+}=\sum_{ij}w_{ij}^{+}$ and all negative connections is $v^{-}=\sum_{ij}w_{ij}^{-}$. The strength of the node i is $s_{i}^{\pm }=\sum_{j}w_{ij}^{\pm }$. The chance-expected within-module connection weight is $e_{ij}^{+}=\frac{s_{i}^{+}s_{j}^{+}}{v^{+}}$ for positive connections and $e_{ij}^{-}=\frac{s_{i}^{-}s_{j}^{-}}{v^{-}}$ for negative connections. $\delta_{M_{i}M_{j}}$ is 1 when i and j are in the same module and 0 otherwise. The quality index is defined as $Q=\frac{1}{v^{+}}\sum_{ij}\left(w_{ij}^{+}-e_{ij}^{+}\right)\delta_{M_{i}M_{j}}-\frac{1}{v^{+}+v^{-}}\sum_{ij}\left(w_{ij}^{-}-e_{ij}^{-}\right)\delta_{M_{i}M_{j}}$

#### Comparison Analysis Using Principal Components

Typically, factor analysis is based on the correlation or covariance between measures, for example, scales of the questionnaire. The correlation or covariance matrix is then decomposed into matrices that explain the maximum amount of variance (principal component analysis) or that are statistically independent (independent component analysis) using singular value decomposition. These methods provide insight into the relationship between the measures, for example, scales loading onto a common factor that is aligned with a theoretical construct. In contrast, the network science approach used in the current study investigates correlations across scales between individuals. It is concerned with the similarity between individuals rather than questionnaire scales. One of the advantages of using the community clustering approach is that individuals get assigned to groups. In contrast, applying a factor analysis would distribute loading on the same individual, which is difficult to interpret.

For comparison with the community clustering results, an exploratory factor analysis using principal component analysis was carried out on the scales of the Conners 3 questionnaire. This analysis was carried out using the psych package v1.7.8 (http://www2.uaem.mx/r-mirror/web/packages/psych/) under R v3.4.1. Varimax rotation was used to obtain orthogonal factors. Inspection of the eigenvalues indicated that a 2-factor solution is sufficient to explain a maximum amount of variance (see scree plot in Figure S3, available online). Using 2 factors, the factor with high loading on Hyperactivity/Impulsivity, Aggression, and Peer Relationships, and a factor with high loading on Inattention, Learning Problems, and Executive Function was found. Using a 3-factor solution, the PCA solution was similar to the dimensions identified using community clustering (Figure S3). The 3-factor PCA showed a factor with high loading on Inattention, Hyperactivity/Impulsivity, and Executive Function, a second factor with high loading on Aggression and Peer Relationships, and a third factor with high loading on Learning Problems. Although this approach is helpful to understand how scales relate to each other and may be related to theoretical constructs, it is difficult to group children based on these results.

#### Structural Connectome Construction

The white matter connectome reconstruction followed the general procedure of estimating the most probably white matter connections for each individual, and then obtaining measures of fractional anisotropy (FA) between regions (Figure 2). The details of the procedure are described in the next paragraphs, and followed the same procedure as previously used.^2^ In the current study, MRI scans were converted from the native DICOM to compressed NIfTI-1 format (dcm2nii). Subsequently, a brain mask was derived from the b0-weighted volume of the diffusion-weighted sequence, and the entire sequence was submitted for correction for participant movement and eddy current distortions through the FMRIB Software Library (FSL) eddy tool. Next, nonlocal means that de-noising^3^ was applied using the Diffusion Imaging in Python (DiPy) v0.11 package^4^ to boost signal-to-noise ratio. The diffusion tensor model was fitted to the pre-processed images to derive maps of fractional anisotropy (FA) using *dtifit* in FSL v.5.0.6.^5^ A constant solid angle (CSA) model was fitted to the 60-gradient-direction diffusion-weighted images using a maximum harmonic order of 8 with DiPy. Next, probabilistic whole-brain tractography was performed based on the CSA model with 8 seeds in any voxel with a General FA value higher than 0.1. The step size was set to 0.5, and the maximum number of crossing fibers per voxel was set to 2.

For the region of interest (ROI) definition, T1-weighted images were preprocessed by adjusting the field of view using FSL *robustfov*; nonlocal means denoising in DiPy, deriving a robust brain mask using the brain extraction algorithm of the Advanced Normalization Tools (ANTs) v1.9 (see Avants *et al.*),^6^ and submitting the images to recon-all pipeline in FreeSurfer v5.3 (http://surfer.nmr.mgh.harvard.edu). Regions of interests (ROIs) were based on the Desikan-Killiany parcellation of the MNI template^7^ with 34 cortical ROIs per hemisphere and 17 subcortical ROIs (brainstem, and bilateral cerebellum, thalamus, caudate, putamen, pallidum, hippocampus, amygdala, and nucleus accumbens). The surface parcellation of the cortex was transformed to a volume using the aparc2aseg tool in FreeSurfer. Furthermore, the cortical parcellation was expanded by 2 mm into the subcortical white matter using in-house software. To move the parcellation into diffusion space, a transformation based on the T1-weighted volume and the b0-weighted image of the diffusion sequence was calculated using FreeSurfer *bbregister* and applied to the volume parcellation. For each pairwise combination of ROIs, the number of streamlines intersecting both ROIs was estimated and transformed to a density map. A symmetric intersection was used; that is, streamlines starting and ending in each ROI were averaged. The weight of the connection matrices was based on fractional anisotropy (FA). To obtain FA-weighted matrices, the streamline density maps were binarized after thresholding, multiplied with the FA map, and averaged over voxels to obtain the FA value corresponding to the connection between the ROIs. This procedure was implemented in-house based on DiPy v0.11 functions.^4^

## Supplementary Analyses

### Comparison Analysis With an Alternative Community Detection Algorithm

The results of the clustering using an alternative community detection algorithm were tested. The Kerighan−Lin algorithm was used for the comparison,^8^ which can incorporate signed edges like the algorithm used in the main analysis. The results indicated that community clustering used in the main analysis and the Kernighan−Lin algorithm produced identical results (Figure S1). This was reflected in the partition distance metrics for the community affiliations produced by the algorithms (normalized variance: 0; normalized mutual information: 1; calculated using Brain Connectivity Toolbox functions).

### Robustness of the Consensus Clustering Algorithm

To test the reliability of the community detection algorithm under varying conditions, random networks with known community structure were created. The networks consisted of 100 nodes with 4 modules. The connection likelihood within and between clusters was systematically varied between 0.1 and 0.9. The quality index of the community structure was calculated at each combination of between- and within-cluster connection likelihood. The results indicated a high-quality index for network with higher within-cluster than outside-cluster connection likelihood (Figure S2a). High connection density outside of clusters had a large influence, even when the connection likelihood within modules was very high.

For comparison with the empirical network of Conners 3 score correlations, the connection density within and between networks was calculated. To this end, all connections were binarized so that any connection with a Pearson correlation coefficient above 0 was set to 1. The connection density was estimated as the ratio between existing connections in the binarized empirical network and a fully connected network of the same size. Connection density within modules based on consensus clustering was 0.79 and connection density between modules was 0.05. Together with the results of the simulated networks, these connection densities indicate very high separation of the network clusters.

We further tested the robustness of the community assignment by adding increasing percentages of random Gaussian noise (*μ* = 0, *σ* = 1) to the network matrix and repeated the consensus clustering procedure (Figure S2a). The quality index indicated good separation of the clusters between 5% and 30% noise (Q between 0.62 and 0.65). No stable assignment could be reached at 35% of noise and above. These results indicate that the community assignment is robust to a considerable amount of noise.

### Influence of Connection Weight and Thresholding on Structural Connectome Results

Different methods exist for the construction of structural networks from diffusion-weighted data, and there is currently no scientific consensus on the best approach.^9^ Networks in the current analysis were weighted by fractional anisotropy (FA), a commonly used measure of white matter organization based on the diffusion tensor model. FA characterizes the directedness of diffusion within a voxel, but may lead to misinterpretation in regions of crossing fibers.^10^ Therefore, the main analysis was repeated with networks weighted by Generalized FA (GFA) based on a constant solid angle (CSA) model, which is better able to take crossing fibers into account.^11^ The node degree for each brain region was identical for the GFA and FA model for density thresholds between 5% and 15% (Kolmogorov−Smirnov test for 2 samples: *p* = 1.0 uncorrected for all regions). It follows that the PLS analysis provides the same results for networks weighted by FA and GFA, as this analysis was based on node degrees, and the node degrees were identical for both models within the relevant density range.

Another potential source of variation in the analysis is the density threshold. Network analyses are sensitive to the number of connections. Therefore, density thresholding is often applied, but the chosen threshold may influence the results of the analysis. For the current investigation, the influence of different density thresholds was systematically investigated by repeating the analysis over a range of densities and comparing the factor scores in a repeated-measures analysis of variance model with factors for density and the interaction between density and component (components loading density + component + density × component). The results indicated no significant effect of density or the interaction between density and any component (model fit: *F*(9, 14790) < 0.001, *p* = 1, adjusted *R*^2^ = −0.001; density: *t <* 0.001, p = 1, interactions: *t <* 0.001, *p* = 1).
